# Comparison of Interferon-Gamma Release Assay and Tuberculin Skin Test in Screening for Latent Tuberculous Infection Among Students from High-Burden Areas: A Prospective Head-to-Head Study in Qingdao, China

**DOI:** 10.3390/tropicalmed10110311

**Published:** 2025-10-31

**Authors:** Zhongdong Wang, Kun Zhang, Haiyan Sun, Xuekui Li, Song Song, Meng Chen, Honghong Xu, Huaqiang Zhang, Yu Pang, Xiaoqi Dai

**Affiliations:** 1 Qingdao Prefectural Center for Disease Control and Prevention, Qingdao Institute of Prevention Medicine, Qingdao 266000, China; stdyq@163.com (Z.W.); 2003-sunhaiyan@163.com (H.S.); qdlxk@outlook.com (X.L.); fzdongjiang@163.com (S.S.); dreamchen0101@163.com (M.C.); alicemenghan1@163.com (H.X.); zhanghq@yeah.net (H.Z.); 2 Beijing Key Laboratory for Key Technologies in Tuberculosis Prevention and Control, Department of Bacteriology and Immunology, Beijing Chest Hospital, Capital Medical University/Beijing Tuberculosis and Thoracic Tumor Research Institute, Beijing 101149, China; kunzhanggr@163.com

**Keywords:** latent tuberculosis infection, tuberculin skin test, interferon-gamma release assays, students, high tuberculosis burden areas

## Abstract

Background: Identifying latent tuberculosis infection (LTBI) is critical for pediatric TB control in China, especially among students from high-burden areas. With no gold-standard test, we compared the tuberculin skin test (TST) and interferon-gamma release assay (IGRA), focusing on factors related to test discordance. Materials and Methods: TST was administered to 1047 local and 900 migrant students; all migrants also received IGRA. TST cutoffs of 5 mm and 10 mm were applied. Agreement was measured using Cohen’s Kappa, and determinants of discordance were analyzed with binary logistic regression. Results: Migrant students had higher TST positivity than locals (28.89% vs. 19.67%, *p* < 0.001). The agreement between IGRA and TST-12 mm (k = 0.491) was higher than that observed for TST-10 mm (k = 0.466) and TST-5 mm (k = 0.356). Subgroup analyses across sex, residence, ethnicity, BMI, TB contact, and BCG history confirmed superior consistency for TST-12 mm. Individuals without BCG vaccination were less likely to show discordance between IGRA and TST-12 mm (OR = 0.32, 95% CI: 0.10–0.81). Conclusions: Using a 12 mm cutoff improves TST accuracy for students from high-burden areas. IGRA should be preferred for individuals with BCG vaccination history.

## 1. Introduction

According to the 2024 Global Tuberculosis Report by the World Health Organization (WHO), global tuberculosis incidence has rebounded in recent years [1]. Latent tuberculosis infection (LTBI) is characterized by a persistent immune response to Mycobacterium tuberculosis (MTB) antigens without the clinical manifestations of active tuberculosis [2]. It is estimated that about a quarter of the global population is in a state of latent infection [3]. China bears the heaviest LTBI burden, with approximately 350 million people living with the infection [2]. Therefore, early identification and intervention for LTBI are crucial strategies for tuberculosis prevention and control.

As highly congregated environments, schools are particularly susceptible to tuberculosis (TB) outbreaks among students. The reported incidence of TB in schools constitutes 4–6% of the total reported cases in China [4]. An analysis of the data reported TB cases in China from 2006 to 2020 revealed that there was a peak in the incidence rate of TB among young adults [5]. Consequently, it is crucial to implement LTBI screening in high schools [6,7]. Additionally, new students recruited by schools come from all over the country, with different infection statuses. Research indicates a notable regional disparity in the percentage change in tuberculosis incidence in China in recent years. Specifically, Tibet and Xinjiang, both located in western China, reported the highest increases, at 124.24% and 114.72%, respectively. In contrast, Shandong Province, where Qingdao City is located, showed a decrease of 15.87% [8]. Therefore, there is a compelling need to focus on the prevalence of LTBI among migrant students from western regions attending schools in Qingdao City. By effectively screening students at high risk of TB infection, we could substantially enhance the efficiency of TB prevention and control.

Currently, there is no universally accepted “gold standard” for diagnosing LTBI. The tuberculin skin test (TST) and interferon-gamma release assays (IGRA) are commonly used worldwide, and both are based on the host’s cellular immune response [9]. Unlike IGRA, which relies on a single cutoff value to indicate MTB infection, TST requires the selection of different cutoff values depending on specific population characteristics, thereby enhancing the accuracy of test results [10]. Both IGRA and TST have their respective technical advantages and limitations, and their performance varies across different regions and populations [11]. Simultaneously, due to the higher cost of IGRA and the requirement for well-equipped laboratories, TST is more suitable for large-scale population screening. Therefore, it is crucial to evaluate the consistency of both methods in diagnosing LTBI in specific populations and to determine the optimal TST cutoff value.

Given the lack of research in China on the diagnosis of LTBI among students in high-burden areas, this study aimed to assess the concordance between different TST cutoff values and IGRA in diagnosing LTBI in this population, to determine the optimal TST cutoff value, and to identify risk factors associated with the inconsistency between the optimal TST cutoff and IGRA results.

## 2. Materials and Methods

### 2.1. Study Population

This study employed a simple cluster sampling design to ensure sample representativeness and research feasibility. First, all high schools in Qingdao City that admitted students from western regions were defined as primary sampling units. From this pool, three high schools were randomly selected without replacement using a simple random sampling. Subsequently, all eligible freshmen from these three selected high schools were included in the study sample, including both local students and migrant students from western areas in 2023. This study chose to screen freshmen because it allowed for the identification of tuberculosis patients or individuals with latent infection before matriculation, thereby achieving precise prevention and control. Furthermore, it helped avoid the confounding effects of the local environment and lifestyle habits on migrant students within the freshman population. Local students had been residing in Qingdao, a low-burden area for tuberculosis, while migrant students came from high-burden areas (Tibet and Xinjiang) in the west but had recently moved to Qingdao to attend school. Consequently, migrant students might be a high-risk group for latent tuberculosis infection. In our study, 1047 local students and 904 migrant students from western areas were included. The exclusion criteria for the study were (1) individuals with a history of tuberculosis or current tuberculosis; (2) individuals with a history of treatment for LTBI or those currently receiving anti-tuberculosis treatment; (3) individuals with contraindications to TST, including allergies to tuberculin components, recent receipt of a live vaccine within the past 4–6 weeks, and those in an immunosuppressive state. Ultimately, 1047 local students and 900 migrant students from western areas were included in the initial TST screening. Since this study focused on migrant students, after verifying the difference in positivity rates between local and migrant students using TST, IGRA testing and questionnaire surveys were conducted among the migrant students to assess diagnostic concordance. Participants who tested positive for LTBI on both tests were referred for preventive treatment.

### 2.2. Questionnaire and Physical Examination

This study collected epidemiological data from migrant students in western areas through a designed questionnaire, including sex, household registration, race, TB contact history, previous diagnosis and screening, and BCG vaccination history. The questionnaire was self-administered, which is explained in more detail in an additional file (see Supplementary Materials File S1). The designated medical institutions for tuberculosis performed physical examinations, screening for suspected tuberculosis symptoms, TST, IGRA, and chest X-ray examinations according to the standards of the “Guidelines for Tuberculosis Prevention and Control in Chinese Schools (2020 Edition)” [12].

### 2.3. TST

Students from local and western areas were all tested with TST using a BCG purified protein derivative (50 IU/mL, 1 mL/vial) from Beijing Xiangrui Biological Products Co., Ltd. (Beijing, China). The test results were assessed by measuring the average diameter of subcutaneous nodules at 72 h. There are multiple categories for a positive TST based on the size of the nodules. An induration <5 mm is interpreted as a negative TST result; nodules with an average diameter of ≥5 mm and <10 mm are generally positive; nodules with an average diameter of ≥10 mm and <15 mm are moderately positive; nodules with an average diameter of ≥15 mm are strongly positive [10]. Different cutoff values are used for the diagnosis of LTBI in different populations. Currently, the widely used TST cutoff values for defining latent tuberculosis infection are 5 mm and 10 mm [13,14]. However, the presence of false-positive results limits its clinical utility. This study aimed to evaluate the concordance between IGRA positivity and multiple TST cutoff points (5 mm, 10 mm, 11 mm, 12 mm, 13 mm, 14 mm, and 15 mm) to determine the optimal TST threshold for identifying LTBI among students in high-burden areas of China.

### 2.4. IGRA Performance

Migrant students from western areas underwent the IGRA test using the Mycobacterium tuberculosis-specific cellular immune response detection kit (enzyme-linked immunosorbent assay) (QuantiFERON-TB Gold, produced by Qiagen, Hilden, Germany). For this test, 5 mL of peripheral venous blood was collected and incubated in a blood culture tube for 16 to 24 h. After incubation, the plasma was extracted and the concentration of γ-interferon produced in response to Mycobacterium tuberculosis-specific antigen stimulation was measured. Results were interpreted qualitatively as positive, negative, or indeterminate based on the manufacturer’s guidance: a test was considered positive when (TB antigen − Nil) ≥ 0.35 IU/mL and ≥25% of the Nil value; negative when both antigen − Nil values were < 0.35 IU/mL (or <0.35 IU/mL but <25% of Nil) with an adequate mitogen control (mitogen − Nil ≥ 0.5 IU/mL); and indeterminate when the Nil value was >8.0 IU/mL or when the mitogen control response was low (mitogen − Nil < 0.5 IU/mL) while antigen responses were negative. Samples with indeterminate IGRA results were retested.

### 2.5. Statistical Analysis

EPIDATA 3.1 was used for double entry of questionnaires. Excel 2016 software was used for data processing. The objective of this study was to evaluate the application performance of IGRA and TST within the target population. The required sample size was estimated using PASS 2021 software to assess the agreement between TST and IGRA results. Based on the previous literature, a kappa (k) value of 0.52 was expected, with a null hypothesis value of k_0_ = 0.40 [15]. Assuming a two-sided significance level (α) of 0.05, statistical power (1 − β) of 0.80, and a distribution of positive and negative results of 0.8:0.2, the minimum required sample size was calculated to be 673 participants. Considering an anticipated 10% loss to follow-up, the final target sample size was set at approximately 740 participants. Statistical analyses were performed using R software (version 4.3.3). Categorical variables were summarized as frequencies and percentages and were tested using the chi-square test. Cohen’s kappa coefficient (k) was used to evaluate the agreement between IGRA and TST results, with k values classified as slight (k < 0.20), fair (0.21 < k < 0.40), moderate (0.41 < k < 0.60), substantial (0.61 < k < 0.80), and almost perfect (0.81 < k < 1.0) [16]. Logistic regression analysis was conducted to identify factors associated with discordance between IGRA and TST results (TST-12 mm cutoff). The dependent variable was whether the results of IGRA and TST-12 mm were inconsistent. Variables with *p* < 0.20 in the univariable analysis and considered clinically relevant were included in the multivariable logistic regression model (six variables in total). Multicollinearity among independent variables was assessed using variance inflation factors (VIFs), and no significant collinearity was detected (all VIF < 5). In addition, a *p*-value of <0.05 was used to define statistical significance in all analyses.

## 3. Results

In our study, 1047 local students and 900 migrant students from western areas were screened by TST. The rate of positive TST results when using the cutoff of 5 mm was 15.18% (159/1047) and 28.89% (260/900) in local students and migrant students, respectively (χ^2^ = 53.81, df = 1, *p* < 0.001). When designated as positive using a 10 mm cutoff, the rate in migrant students was 19.67% (177/900), which was also significantly higher than that in local students, at 11.65% (122/1047) (χ^2^ = 23.91, df = 1, *p* < 0.001). Comparative analysis revealed significantly higher TST positivity rates in the migrant student population, identifying them as a high-risk group. Therefore, this study focused on the application performance and discordance factors of IGRA and TST in migrant students from western areas, which have a higher burden of tuberculosis.

The main characteristics of migrant students from western areas (n = 900) as assessed by IGRA and TST were shown in Table 1. There was no difference in household registration among TST ≥ 5 mm, TST ≥ 10 mm, and IGRA positivity. Compared with Han participants, participants of other races had significantly higher positivity on IGRA (*p* = 0.007). For TST ≥ 10 mm, positivity also differed by BMI group (*p* = 0.028) and sex (*p* = 0.044). The rate of TST positivity showed a significant increase in students with prior TB contact (*p* < 0.001). Similarly, students with BCG vaccination had higher positivity on TST ≥ 10 mm and IGRA.

Utilizing Cohen’s kappa coefficients, the agreement between the IGRA and TST results were evaluated and are shown in Table 2. The level of agreement between IGRA and TST-5 mm was 0.356, with a concordant rate of 76.78%, indicating fair agreement (*p* < 0.001). For the 10 mm cutoff, the kappa value was 0.466, with a concordance rate of 84.00%, representing moderate agreement (*p* < 0.001). The highest agreement was observed at the 12 mm cutoff, with a kappa value of 0.491 and a concordance rate of 86.33%, also indicating a moderate agreement (*p* < 0.001). Therefore, the agreement between TST-12 mm and IGRA was higher than that observed for the commonly used 5 mm and 10 mm TST cutoffs.

We further compared the agreement between IGRA and TST-5 mm, TST-10 mm, and TST-12 mm across different subgroups. As indicated by the results in Table 3, IGRA and TST-5 mm presented a moderate agreement in patients who were female (k = 0.410, *p* < 0.001), and from rural areas (k = 0.440, *p* < 0.001) but showed slight to fair agreement in the remaining variables. Urban residence (k = 0.342, *p* < 0.001), Han ethnicity (k = 0.322, *p* < 0.001), and BMI ≥ 24 (k = 0.341, *p* < 0.001) were variables that presented fair agreement between IGRA and TST-10 mm results, with the rest of the variables associated with moderate agreement. The agreement between IGRA and TST-12 mm was fair among males, participants of Han ethnicity, and participants with BMI ≥ 24, substantial among those with a history of TB contact, and moderate for the remaining variables. Overall, the concordance of IGRA with TST-10 mm and TST-12 mm was higher than that with TST-5 mm across all subgroups. Compared with IGRA and TST-10 mm, the concordance of IGRA and TST-12 mm was slightly lower in males, rural residents, participants of Han ethnicity, and participants with BMI ≥ 24, but was the highest for all other variables. The detailed distribution of discordant cases for each subgroup is presented in Supplementary Materials File S2. Therefore, a cutoff value of 12 mm was identified as having the highest diagnostic value for TST in this population. The factors affecting the discordance between IGRA and TST-12 mm are worthy of further research.

Table 4 showed the risk of discordance between IGRA and TST-12 mm by univariable analysis. The discordance of TST−/IGRA+ was higher than TST+/IGRA−. The differences in the distribution of the discordance between IGRA and TST-12 mm across various variables were not statistically significant (*p* > 0.05). Individuals without BCG vaccination had a significantly less likelihood of discordant results between IGRA and TST-12 mm (OR 0.32; 95% CI 0.10, 0.81).

**Figure 1:**
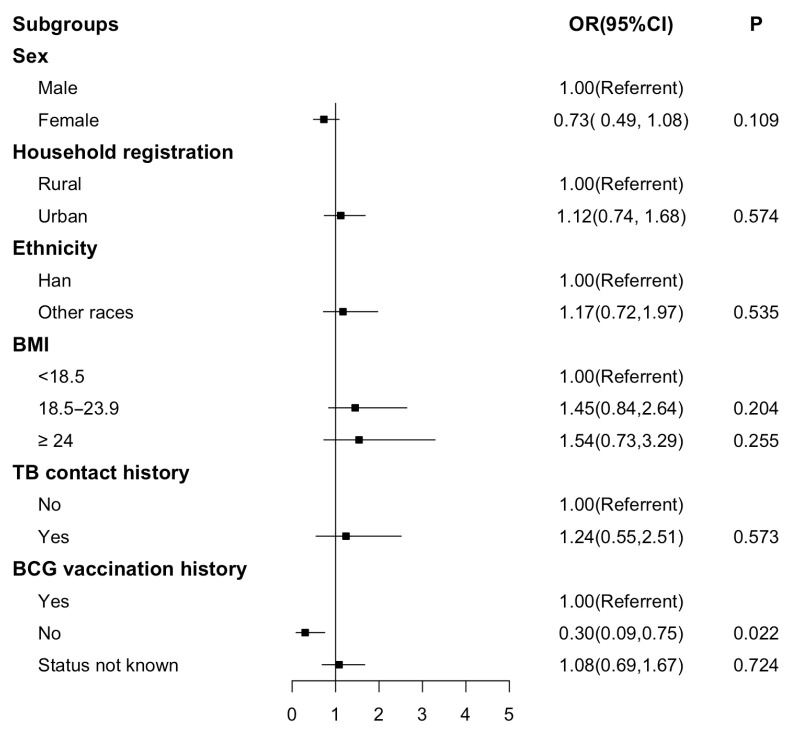
Forest plot for multivariable analysis of the discordance between IGRA and TST-12 mm. Figure legends: Forest plot for performance on the odds ratio of discordance between IGRA and TST-12 mm. A logistic regression model was used. OR, odds ratio; CI, confidence interval.

Further, a significant difference was still retained after adjustment (Figure 1). Students who had not received the BCG vaccination were less likely to show discordance between TST and IGRA results (OR 0.30; 95% CI 0.09, 0.75).

## 4. Discussion

As China is a country with a high burden of tuberculosis, it is imperative to focus on students at high risk of tuberculosis infection in congregate settings such as schools. However, there are few studies on students from areas with a high burden of tuberculosis in China. Consequently, there is an urgent need to establish an effective LTBI detection system. Such a system would not only advance the threshold of TB prevention and control and reduce the incidence of tuberculosis but also lay the foundation for studying the LTBI epidemic characteristics in students from high-burden areas. Therefore, by evaluating the effectiveness of IGRA and TST among students in high-burden areas, this study aimed to determine the optimal TST cutoff value and propose a localized LTBI detection system specifically for this population.

Western areas in China are high-burden regions for tuberculosis. This study conducted TST screening on 1047 local students and 900 migrant students from western areas and found that the positive rate was higher among those students. Collins et al. have confirmed that the detection rate of LTBI is positively correlated with the incidence of tuberculosis [17]. Early screening can detect latent infections as early as possible, which can effectively avoid the occurrence of clustered epidemics. In addition, after analyzing the IGRA and TST results of the 900 students, it was found that the IGRA positivity rate in students of other races was higher than in Han students. This might be explained by the fact that students of other races are mostly from rural areas or small towns, which have differences in terms of medical and health service systems compared to areas dominated by Han populations. Moreover, the BCG vaccination rate was low, which could significantly increase the disease burden in rural areas [18]. Our study also showed that the TST-positive rate of students with TB contact history was higher than that in students without TB contact history. Some studies are consistent with our results [19]. This study revealed that the TST-positive rate of students with BCG vaccination history was higher than that of those without BCG vaccination history. One study has shown that TST is more sensitive than IGRA for screening LTBI in the BCG-vaccinated population, although it is less specific [20].

In this study, we observed a moderate agreement between IGRA and TST-12 based on kappa coefficients, which was better than the agreement obtained for TST-10 and TST-5. This finding was not entirely consistent with some previously published studies and existing guidelines. Farhat et al. found that using 10 mm instead of 5 mm as the critical value for diagnosing LTBI reduced the false positive rate [21]. In the Chinese guidelines for the two-step TST–IGRA testing strategy, 5 mm and 10 mm cutoffs were recommended for different populations [14]. However, Erol et al. found that the false positive rate of TST decreased as the critical value increased [22]. Lu et al. also found that when 10–12 mm was selected as the critical value for TST to diagnose LTBI, its diagnostic value was the highest and the specificity was >80% [23]. Consistent with these findings, our study demonstrated that adopting a 12 mm cutoff among students from high-TB-burden areas improved the concordance between TST and IGRA in diagnosing LTBI.

Through univariable and multivariable analysis, our study found that the probability of discordance between IGRA and TST-12 mm decreased in students who had no BCG vaccination history. BCG vaccination may affect TST results. A Canadian study showed that the consistency between IGRA and TST was better in people who had not been vaccinated with BCG, and the authors believed that IGRA was more suitable for people with a history of BCG vaccination [24]. The WHO has also pointed out that IGRA does not contain antigens that cross-react with BCG and has better specificity [25]. Therefore, we suggest that if students from high-burden areas have a clear history of BCG vaccination, IGRA screening for LTBI could be considered directly.

There were several limitations to our study to be considered. First, lifestyle habits, latent NTM infection, and comorbidities such as smoking, diabetes, HIV, and so on were not included in the variables. Second, because the enrolled participants were migrant students from western areas in Qingdao, the population in the study was not entirely representative of the general population or all students in western areas. However, it is worth noting that the strength of this study lies in its potential to provide valuable guidance. The results are expected to facilitate efficient, widespread screening of students at elevated infection risk while concurrently contributing new perspectives to school-based tuberculosis prevention and control strategies.

## 5. Conclusions

Among students from high-tuberculosis-burden areas in western China, IGRA showed better consistency with TST when using a 12 mm cutoff compared to 5 mm and 10 mm thresholds for screening LTBI. This compelling evidence strongly supports setting the TST-positive cutoff at 12 mm for this specific population. Additionally, for individuals with a history of BCG vaccination, it is advisable to preferentially utilize IGRA over TST, ensuring a more accurate and dependable screening process.

## Figures and Tables

**Table 1:** Characteristics of migrant students from western areas by IGRA and TST.

Variables	Subjects, n (%) (N = 900)	TST Positivity (≥5 mm)		TST Positivity (≥10 mm)		IGRA Positivity ((TB Antigen − Nil) ≥ 0.35 and ≥25% of Nil) ^†^	
		n (%)	*p* *	n (%)	*p* *	n (%)	*p* *
Sex			0.484		0.044		0.317
Male	344 (38.22)	104 (30.23)		56 (22.95)		53 (15.41)	
Female	556 (61.78)	156 (28.06)		121 (21.76)		100 (17.99)	
Household registration			0.297		0.394		0.236
Urban	326 (36.22)	101 (30.98)		69 (21.17)		49 (15.03)	
Rural	574 (63.78)	159 (27.70)		108 (18.82)		104 (18.12)	
Race			0.347		0.198		0.007
Han	184 (20.44)	48 (26.09)		30 (16.40)		19 (10.33)	
Other races	716 (79.56)	212 (29.61)		147 (20.53)		134 (18.72)	
BMI			0.137		0.028		0.378
<18.50	151 (12.78)	34 (22.52)		21 (13.91)		25 (16.56)	
18.5–23.99	644 (71.56)	197 (30.59)		141 (21.89)		115 (17.86)	
≥24	105 (11.67)	29 (27.62)		15 (14.29)		13 (12.38)	
TB contact history			<0.001		<0.001		0.176
No	845 (93.89)	228 (26.98)		151 (17.87)		140 (16.57)	
Yes	55 (6.11)	32 (58.18)		26 (47.27)		13 (23.64)	
BCG vaccination history			0.650		0.042		0.033
No	78 (8.67)	19 (24.36)		7 (8.97)		5 (6.41)	
Yes	609 (67.67)	178 (29.23)		128 (21.02)		110 (18.06)	
Status not known	213 (23.67)	63 (29.58)		42 (19.72)		38 (17.84)	

* χ^2^ test for the difference (*p* < 0.05) between the groups. ^†^ Nil: the unstimulated negative control tube.

**Table 2:** Agreement between IGRA and TST (TST-5, 10, 11, 12, 13, 14, and 15 mm).

TST Cutoff	TST−/IGRA− n (%)	TST+/IGRA+ n (%)	TST−/IGRA+ n (%)	TST+/IGRA− n (%)	Kappa	*p*	Concordance (%)
TST-5 mm	589 (65.44)	102 (11.33)	51 (5.67)	158 (17.56)	0.356	<0.001	76.78
TST-10 mm	663 (73.67)	93 (10.33)	60 (6.67)	84 (9.33)	0.466	<0.001	84.00
TST-11 mm	687 (76.33)	84 (9.33)	69 (7.67)	60 (6.67)	0.480	<0.001	85.67
TST-12 mm	695 (77.22)	82 (9.11)	71 (7.89)	52 (5.78)	0.491	<0.001	86.33
TST-13 mm	708 (78.67)	74 (8.22)	79 (8.78)	39 (4.33)	0.482	<0.001	86.89
TST-14 mm	714 (79.33)	71 (7.89)	82 (9.11)	33 (3.67)	0.481	<0.001	87.22
TST-15 mm	717 (79.67)	63 (7.00)	90 (10.00)	30 (3.33)	0.440	<0.001	86.67

**Table 3:** Agreement between IGRA and TST (TST-5 mm, TST-10 mm, and TST-12 mm) among different subgroups.

Variables	IGRA and TST-5 mm			IGRA and TST-10 mm			IGRA and TST-12 mm		
	Kappa	*p*	Concordance (%)	Kappa	*p*	Concordance (%)	Kappa	*p*	Concordance (%)
Sex									
Male	0.272	<0.001	73.55	0.401	<0.001	84.30	0.354	<0.001	84.59
Female	0.410	<0.001	78.78	0.499	<0.001	83.99	0.559	<0.001	87.41
Household registration									
Urban	0.214	<0.001	71.17	0.342	<0.001	80.37	0.424	<0.001	85.28
Rural	0.440	<0.001	79.97	0.537	<0.001	86.06	0.526	<0.001	86.93
Race									
Han	0.212	<0.001	75.54	0.322	<0.001	84.24	0.309	<0.001	87.50
Other races	0.385	<0.001	77.09	0.491	<0.001	83.94	0.516	<0.001	86.03
BMI									
<18.5	0.393	<0.001	80.79	0.539	<0.001	88.08	0.560	<0.001	89.40
18.5–23.9	0.371	<0.001	76.40	0.465	<0.001	82.92	0.500	<0.001	85.87
≥24	0.196	0.024	73.33	0.341	<0.001	84.76	0.298	<0.001	84.76
TB contact history									
No	0.350	<0.001	77.51	0.456	<0.001	84.50	0.473	<0.001	86.51
Yes	0.364	<0.001	65.45	0.513	<0.001	76.36	0.618	<0.001	83.64
BCG vaccination history									
No	0.165	0.055	76.92	0.460	<0.001	92.30	0.573	<0.001	94.87
Yes	0.392	<0.001	77.67	0.468	<0.001	83.25	0.494	<0.001	85.88
Status not known	0.300	<0.001	74.18	0.446	<0.001	83.10	0.455	<0.001	84.51

**Table 4:** Univariable analysis of the discordance between IGRA and TST-12 mm.

	Discordance		Risk of Discordance (Unadjusted)	
	N (%)	*p* *	OR (95% CI)	*p* ^†^
TST+/IGRA−	52 (5.78)			
TST−/IGRA+	71 (7.89)			
Sex		0.189		
Male	54 (15.70)		1.00 (Referent)	
Female	70 (12.59)		0.77 (0.53, 1.14)	0.189
Household registration		0.411		
Rural	75 (13.07)		1.00 (Referent)	
Urban	49 (15.03)		1.17 (0.79, 1.73)	0.412
Race		0.746		
Han	24 (13.04)		1.00 (Referent)	
Other races	100 (13.97)		1.08 (0.68, 1.78)	0.746
BMI		0.446		
<18.5	16 (10.60)		1.00 (Referent)	
18.5–23.9	92 (14.29)		1.41 (0.82, 2.55)	0.235
≥24	16 (15.24)		1.52 (0.72, 3.21)	0.272
TB contact history		0.566		
No	115 (13.61)		1.00 (Referent)	
Yes	9 (16.36)		1.24 (0.56, 2.49)	0.567
BCG vaccination history		0.062		
Yes	87 (14.29)		1.00 (Referent)	
No	4 (5.13)		0.32 (0.10, 0.81)	0.032
Status not known	33 (15.49)		1.10 (0.70, 1.68)	0.668

* χ^2^ test for the difference (*p* < 0.05) between the groups. ^†^ Simple logistic regression model, OR odds ratio, CI confidence interval.

## Data Availability

The data supporting the findings of this study are not publicly available due to ethical and institutional privacy restrictions but may be made available from the corresponding author upon reasonable request and with appropriate institutional approvals.

## Supplementary Materials

The following supporting information can be downloaded at https://www.mdpi.com/article/10.3390/tropicalmed10110311/s1, File S1: Questionnaire, File S2: Table S1. Distribution of discordant cases among different subgroups.

## Abbreviations

TST	Tuberculin skin test
IGRA	Interferon-gamma release assays
LTBI	Latent tuberculous infection
CI	Confidence interval
WHO	World Health Organization
MTB	Mycobacterium tuberculosis
TB	Tuberculosis
