# Supplementary material for: Comparison of Interferon-Gamma Release Assay and Tuberculin Skin Test in Screening for Latent Tuberculous Infection Among Students from High-Burden Areas: A Prospective Head-to-Head Study in Qingdao, China
Source: Trop Med Infect Dis. 2025 Oct 31;10(11):311. doi: 10.3390/tropicalmed10110311 (PMC12656460; doi:10.3390/tropicalmed10110311)
Supplement: Supplementary file 1 [file tropicalmed-10-00311-s001.zip › File S1.pdf]

Questionnaire

I. Basic Information

1.Name: ________________

2.School: ________________

3.Actual Age: _______ years

4.Ethnicity: _______ ethnic group

5.Pre-admission Household Registration Type: (1) Urban (2) Rural

6.Grade and Class: Grade _______ Class _______

7.Permanent Residence: Province of ________________

8.Gender: (1) Male (2) Female

9.Height: _______ cm

10.Weight: _______ kg

11.Tuberculosis (TB) Symptom Survey (Presence of the following symptoms in the past 6 months, and duration if applicable):

12.Cough: (1) Yes _______ weeks (2) No

13.Sputum Production: (1) Yes _______ weeks (2) No

13.1. Hemoptysis (Coughing up blood): (1) Yes _______ weeks (2) No

13.2. Chest Pain: (1) Yes _______ weeks (2) No

13.3. Night Sweats: (1) Yes _______ weeks (2) No

13.4. Low-Grade Fever: (1) Yes _______ weeks (2) No

13.5. Fatigue: (1) Yes _______ weeks (2) No

13.6. Weight Loss: (1) Yes _______ weeks (2) No

14. Smoking History: (1) Yes (2) No

14.1. How many times do you smoke per week? _______ times

15. Alcohol Consumption History: (1) Yes (2) No

II. Medical History

16. History of Tuberculosis (TB): (1) Yes (Time since last diagnosis: ______ years ______ months) (2) No (3) Don’t know

16.1. How many times have you had TB? _______ □ Don’t know

16.2. Date of most recent onset: _______ year _______ month □ Don’t know

16.3. Was treatment received? (1) Yes (2) No

16.4. Start date of most recent treatment: _______ year _______ month

16.5. End date of most recent treatment: _______ year _______ month □ Currently undergoing treatment

17. Diabetes: (1) Yes (2) No (3) Don’t know

18. Hypertension: (1) Yes (2) No (3) Don’t know

19. Silicosis: (1) Yes (2) No (3) Don’t know

20. Chronic Obstructive Pulmonary Disease (COPD): (1) Yes (2) No (3) Don’t know

21. Respiratory Diseases: (1) Yes (2) No (3) Don’t know

22. Malignant Tumors: (1) Yes (2) No (3) Don’t know

23. Chronic Renal Failure: (1) Yes (2) No (3) Don’t know

24. Hepatitis: (1) Yes (2) No (3) Don’t know

25. Liver Cirrhosis: (1) Yes (2) No (3) Don’t know

26. HIV/AIDS: (1) Yes (2) No (3) Don’t know

27. Long-Term Use of Corticosteroids and/or Immunosuppressants: (1) Yes (2) No (3) Don’t know

III. Lifestyle and TB Exposure History

28. Family History of TB: (1) Yes (2) No (3) Don’t know

28.1. Number of permanent household members: _______ people

29. Have you ever been in contact with someone diagnosed with TB? (1) Yes (2) No (3) Don’t know

30. Co-residence with TB Patient: (1) Yes (Duration of contact: _______ months) (2) No

31. Have you ever been vaccinated with BCG (visible scar on the arm)? (1) Yes (2) No

IV. Tuberculosis Knowledge and Healthcare-Seeking Behavior

32. Have you heard of tuberculosis (TB)? (1) Yes (2) No

32.1. How is TB primarily transmitted?

(1) Through airborne droplets when coughing

(2) Through handshakes

(3) Through sharing food

(4) Through touching objects

(5) Don’t know

32.2. What do you think causes TB?

(1) Bacteria (2) Cold weather (3) Smoking (4) Poor hygiene (5) Don’t know

32.3. How long does TB treatment typically last?

(1) 1-2 weeks (2) 1-2 months (3) 6-8 months (4) Incurable (5) Don’t know

33. To your knowledge, can someone be infected with TB without feeling symptoms?

(1) Yes (2) No

33.1. To your knowledge, is coughing/sputum production for ≥2 weeks, fever, or chest pain a symptom of TB?

(1) Yes (2) No

33.2. To your knowledge, are weight loss and night sweats symptoms of TB?

(1) Yes (2) No

33.3. If you suspected you had TB, would you wait to see if symptoms resolve before seeking medical care?

(1) Yes (2) No

33.4. If a doctor said you might have TB but you had no symptoms, would you seek treatment or wait for symptoms to appear?

(1) Seek treatment (2) Wait for symptoms

34. Should a patient stop taking medication once they feel better?

(1) Yes (2) No (3) Don’t know

34.1. What happens if treatment is stopped prematurely?

(1) Patient recovers (2) Develops drug-resistant TB (3) Don’t know

34.2. Can TB reoccur after recovery?

(1) Yes (2) No (3) Don’t know

34.3. How can TB be prevented? (Multiple choice)

(1) Cover mouth/nose when coughing/sneezing

(2) Exercise regularly

(3) Ventilate indoor spaces

(4) Maintain good nutrition

(5) Don’t know

34.4. What are the risks of not receiving treatment? (Multiple choice)

(1) Death (2) Cure (3) Drug resistance (4) Incurability (5) Don’t know

34.5. How effective do you think non-traditional treatments (e.g., traditional Chinese medicine, herbal therapies, rest, prayer) are for TB?

(1) Very effective (2) Effective (3) Neutral (4) Ineffective (5) Very ineffective

V. Attitudes Toward TB

35. If someone in your community/village/dormitory is diagnosed with TB, how do you think people would react?

(1) Avoid contact, even if it seems unfriendly

(2) Avoid contact but remain polite

(3) Offer support and help

36. Avoiding contact with TB patients to prevent infection is most important.

(1) Yes (2) No

37. People with TB should feel ashamed of their illness.

(1) Yes (2) No

38. I would not want to sit next to a TB patient on public transportation.

(1) Yes (2) No

VI. Psychological Assessment

(Responses for all items below: 1 = Never or rarely; 2 = A little of the time; 3 = A moderate amount of the time; 4 = Almost all or all the time)

39. I feel downhearted and gloomy.

40. I feel best in the morning.

41. I cry or feel like crying.

42. I have trouble sleeping at night.

43. I eat as much as usual.

44. I enjoy close contact with the opposite sex as before.

45. I suffer from constipation.

46. I notice weight loss.

47. My heart races faster than usual.

48. I feel tired for no reason.

49. My mind is as clear as usual.

50. I perform daily tasks without difficulty.

51. I feel restless and unable to calm down.

52. I feel hopeful about the future.

53. I get irritated or angry more easily.

54. Making decisions is easy for me.

55. I feel useful and needed.

56. My life is meaningful.

57. I think others would be better off if I died.

58. I remain interested in things I used to enjoy.

64. I feel more nervous or anxious than usual.

65. I feel afraid for no reason.

66. I feel easily upset or panicked.

67. I feel like I might lose my mind.

68. I feel that everything is fine and no misfortune will happen.

69. My hands or legs tremble.

70. I am troubled by headaches, neck pain, or back pain.

71. I feel weak and fatigued.

72. I feel calm and can sit still easily.

73. I feel my heart pounding.

74. I am troubled by dizziness.

75. I feel faint or like I might faint.

76. I can breathe in and out easily.

77. My hands or feet feel numb or tingly.

78. I am troubled by stomachaches or indigestion.

79. I urinate frequently.

80. My hands are usually dry and warm.

81. My face flushes or feels hot.

82. I fall asleep easily and sleep well through the night.

83. I have nightmares.
