# Supplementary material for: Comparison of Interferon-Gamma Release Assay and Tuberculin Skin Test in Screening for Latent Tuberculous Infection Among Students from High-Burden Areas: A Prospective Head-to-Head Study in Qingdao, China
Source: Trop Med Infect Dis. 2025 Oct 31;10(11):311. doi: 10.3390/tropicalmed10110311 (PMC12656460; doi:10.3390/tropicalmed10110311)
Supplement: Supplementary file 1 [file tropicalmed-10-00311-s001.zip › File S2.pdf]

Table S1. Distribution of discordant cases among different subgroups.

Variables	TST-5mm				TST-10mm				TST-12mm			
	TST-/IGRA- n (%)	TST+/IGRA+ n (%)	TST-/IGRA+ n (%)	TST+/IGRA- n (%)	TST-/IGRA- n (%)	TST+/IGRA+ n (%)	TST-/IGRA+ n (%)	TST+/IGRA- n (%)	TST-/IGRA- n (%)	TST+/IGRA+ n (%)	TST-/IGRA+ n (%)	TST+/IGRA- n (%)
**Sex**												
Male	220(63.95)	33(9.59)	20(5.81)	71(20.64)	262(76.16)	27(7.85)	26(7.56)	29（8.43）	270(78.49)	21(6.10)	32(9.30)	21(6.10)
Female	369(66.37)	69(12.41)	31(5.58)	87(15.65)	401(72.12)	66(11.87)	34(6.12)	55（9.89）	425(76.44)	61(10.97)	39(7.01)	31(5.58)
**Household registration type**												
Urban	204(62.58)	28(8.59)	21(6.44)	73(22.39)	235(72.09)	27(8.28)	22(6.75)	42（12.88）	253(77.61)	25(7.67)	24(7.36)	24(7.36)
rural	385(67.07)	74(12.89)	30(5.23)	85(14.81)	428(74.56)	66(11.50)	38(6.62)	42（7.32）	442(77.00)	57(9.93)	47(8.19)	28(4.88)
**Ethnicity**												
Han	128(69.57)	11(5.98)	8(4.35)	37(20.11)	145(78.80)	10(5.43)	9(4.89)	20（10.87）	154(83.70)	7(3.80)	12(6.52)	11(5.98)
Other races	461(64.39)	91(12.71)	43(6.01)	121(16.90)	518(72.35)	83(11.59)	51(7.12)	64（8.94）	541(75.56)	75(10.47)	59(8.24)	41(5.73)
**BMI(kg/m2)**												
<18.5	107(70.86)	15(9.93)	10(6.62)	19(12.58)	119(78.81)	14(9.27)	11(7.28)	7（4.64）	122(80.79)	13(8.61)	12(7.95)	4(2.65)
18.5-23.9	412(63.98)	80(12.42)	35(5.43)	117(18.17)	461(71.58)	73（11.34）	42(6.52)	68（10.56）	489(75.93)	64(9.94)	51(7.92)	40(6.21)
≥ 24	70(66.67)	7(6.67)	6(5.71)	22(20.95)	83(79.05)	6(5.71)	7(6.67)	9（8.57）	84(80.00)	5(4.76)	8(7.62)	8(7.62)
**History of close contact with TB patients**												
No	566(66.98)	89(10.53)	51(6.04)	139(16.45)	634(75.03)	80(9.47)	60(7.10)	71（8.40）	661(78.22)	70(8.28)	70(8.28)	44(5.21)
Yes	23(41.82)	13(23.64)	0(0.00)	19(34.55)	29(52.73)	13(23.64)	0(0.00)	13（23.64）	34(61.82)	12(21.82)	1(1.82)	8(14.55)
**BCG vaccination history**												

No	57(73.08)	3(3.85)	2(2.56)	16(20.51)	69(88.46)	3(3.85)	2(2.56)	4（5.13）	71(91.03)	3(3.85)	2(2.56)	2(2.56)
Yes	397(65.19)	76(12.48)	34(5.58)	102(16.57)	439(72.09)	68(11.17)	42(6.90)	60（9.85）	464(76.19)	59(9.69)	51(8.37)	35(5.75)
Status not known	135(63.38)	23(10.80)	15(7.04)	40(18.78)	155(72.77)	22(10.33)	16(7.51)	20(9.39)	160(75.12)	20(9.39)	18(8.45)	15(7.04)
